# Round the Hospitals

**Published:** 1921-05-28

**Authors:** 


### ROUND THE HOSPITALS.

The outstanding feature in the financial report of the College of Nursing is the fact that its expenditure is in excess of its income by £748. Sir Arthur Stanley warned members last year that they could not hope for any diminution of expenses in an Association so full of vitality, and his words have come true. The income from interest on investments amounts to £2,742, and this is not nearly enough. To be in a thoroughly sound financial position the College should be able to invest a fixed proportion of all fees for registration and life subscriptions. Every year now will add no doubt to the sum contributed in annual subscriptions, which last year amounted to £422, but members of the College who have its future stability at heart should press forward this year to secure the remaining half of the endowment fund of £100,000 forecasted long ago as necessary for its full development. A concerted movement aiming at an average of £2,000 carried out in every centre of the College would bring about this achievement.

The voting-paper for the Council of the College is the outcome this year of earnest preparatory work all over the kingdom, and may be considered fairly representative of members' views. The English and Welsh section contains the names of thirty-seven matrons, ex-matrons, and superintendents; three medical men; two sister-tutors; one sister-in-charge; four nurses; one health visitor; one midwife; one peeress and one Member of Parliament. Quite evidently nurses who nominate for the Council are persuaded that a nurse does not cease to be fit to regard nursing matters from a sane and well-balanced point of view because she receives promotion. Members should signalise their appreciation of the College *annus mirabilis* by a record poll. Those of the London centre are reminded that as the result of the postal ballot it was decided to support Lady Cowdray, Miss Billing, Miss Amy Hughes, Miss Marsters, and Miss Redl. Certain of the provincial Local Centres have also selected candidates, whose names have been recorded from time to time in

**ROUND THE HOSPITALS**—(*continued*).

these columns, and members before they vote should take the trouble to look them up.

A meeting of the General Nursing Council for England and Wales will be held at 11 a.m. on Friday, June 3, at the Ministry of Health. It is two months since the last meeting was held, and it is probable, therefore, that the coming one will be one of particular interest. We hope that, at any rate, some definite information will be forthcoming as to the sanction of the Registration Rules by the Minister of Health. We understand that the General Nursing Council's new premises at York Gate are on the point of completion, and that the move thither will take place very shortly.

The many friends of Miss Hoadley, Superintendent of the Nurses' Co-operation, will be glad to know that her resignation is due only to private reasons, and would have taken place more than a year ago had she followed her inclinations. She is in robust health and is joining her sister in the conduct of a nursing home out of London. Miss Hoadley, who was trained at Guy's Hospital, has filled the office of Superintendent of the Nurses' Co-operation for ten years, succeeding Mrs. Lucas before the Co-operation moved from New Cavendish Street. It was no easy task to fill Mrs. Lucas' place, but Miss Hoadley quietly took up the reins and under her care the Co-operation has continued its successful career. Her successor has not yet been appointed, and we understand she will have the advantage of Miss Hoadley's assistance until July to gather up the threads. We wish Miss Hoadley the greatest contentment and success in her new work to which the qualities she possesses undoubtedly entitle her.

Lady Astor has done a very kind and practical thing in inviting Plymouth nurses to make use of her drawing-room and verandah overlooking the Hoe three afternoons a week, pending the formation of a nurses' club in the town. There are many nurses as well as others working in Plymouth who feel the need for a club of their own, and a Committee, of which the Viscountess is President, has been formed to promote the movement. There is much to be said in favour of mixed clubs, and the Plymouth Committee is no doubt well advised in proposing to admit other professional women as well.

An alarming state of affairs was revealed at the recent meeting of the Gosport branch of the Victoria Nursing Society. Unless the income can be speedily increased retrenchment must take place, and the present commodious headquarters at Spring Garden Lodge be given up. The Countess of March, in the course of a rousing speech deprecating any backward policy, called attention to the wastage which went on through overlapping, and reported that on one occasion she had found the health visitor, the school nurse, the midwife, and the inspector of midwives all together in the same house. If her advice be taken and greater publicity secured for the work of the Society in Gosport, and economy be practised, not in salaries, but in administration, and the sympathies of every person in the town enlisted, we feel confident next year will find the situation materially improved.

That the Annual Meeting of the College of Nursing is to take place this year in Edinburgh will doubtless prevent many members on this side of the Tyne from being present, especially as it is very uncertain whether the regular train service will be running in a month's time. But it is highly desirable that the unity of the College as a rallying point for nurses in every part of Great Britain should be realised, and the Scottish headquarters represent a most energetic and live section of the members. As a definite festival, in addition to its purposes as a business meeting, the annual gathering promises this year to be unique. It is to take place on Friday, June 24, in the Hall of the Royal College of Surgeons, Edinburgh, at 2.30 p.m., and will be followed by a garden party at the Royal Infirmary in the afternoon and a Nursing Conference at the College of Nursing Club in the evening. For Saturday various excursions of a most enticing character are being arranged, and hospitality is to be secured to members coming from a distance. Members who can manage to take this delightful week-end will find themselves very well looked after indeed.

The Imperial Nurses' Club, at 137 Ebury Street, S.W. 1, continues to advance. It was founded four years and a-half ago, and its sleeping accommodation has grown from seven to thirty beds, nearly always occupied. A new lounge was opened last autumn, the dining-room has been enlarged, and the common rooms include also a fine drawing-room, a writing-room, and one for affording rest and quiet. A cloak-room, with hot and cold water, has recently been added on the ground floor. The Club forms a happy meeting-ground for widely-sundered members of the nursing profession, and as an illustration of the ability of nurses to support a really high-class club it must be considered a pioneer.

Infant welfare workers, whether professional or lay, and in the former is included the medical man or woman, as well as the nurse, and salaried or voluntary, have the opportunity to take part in a competition which the Association of Infant Welfare and Maternity Centres is organising. Two prizes of £5 5s. each are offered respectively for (1) the best syllabus covering one year's educational work in the form of three courses of twelve lectures each; and (2) the text, which must not exceed 1,500 words, of the competitor's favourite health talk on any one of the subjects mentioned in the syllabus. A further interesting feature is that a selection from the best talks will subsequently be published in book form, and the author of each of the published talks will be entitled to share in a 10 per cent. royalty on the sales. The competition closes on June 4, by which date all entries should be received at the Association's offices, 4 and 5 Tavistock Square, W.C.

**ROUND THE HOSPITALS—(*continued*).**

It is really time that a protest was made against the constant cry in the lay papers that "nursing is out of fashion"—a "Cinderella among the professions." Positively a lady writing in the *Liverpool Daily Post* inquires, "Who ever meets a girl who wants to become a nurse?" The answer is, "The matrons of every training-school in the country." Here and there some institution, unhappily placed as regards situation or class of work, vainly appeals for candidates. But within the last twenty years the institutions entitled to rank as training-schools have increased probably by one-third, and within the last seven years the number of candidates annually received for training has gone up by at least 50 per cent.

The District Superintendent of the Cumberland Nursing Association has been subjected to a public attack made upon her by the Secretary of the Kirkbride Nursing Association at the meeting of the County Council. We are glad to see that the Executive Committee at once took action, sifted the whole circumstances with great care, and entirely exonerated the Superintendent, Miss March, at the recent annual meeting. Dr. Morison, the County Medical Officer, commenting on the very satisfactory year's work of the Cumberland Nursing Association, was greeted with applause when he said that the whole responsibility for its being carried out efficiently rested on Miss March, of whose work he could not speak too highly.

Newcastle has begun to economise in the time-honoured manner by cutting down its health service. The health visitors have been reduced in number from twenty to twelve, and Dr. Kerr, the M.O.H., is asking the councillors to reflect that some districts will not be visited at all and some very imperfectly.

Nearly £95,000 has been collected at Nottingham towards the War Memorial Fund for the extension of the General Hospital and the building of a new nurses' home. The annual report announces that the foundations of the home are now nearly completed. The site is on land adjoining St. Peter's Rectory and overlooking the castle grounds. The home will be of a size to give accommodation to the entire nursing staff, now housed in three separate buildings.

